# Application of WES towards Molecular Investigation of Congenital Cataracts: Identification of Novel Alleles and Genes in a Hospital-Based Cohort of South India

**DOI:** 10.3390/ijms21249569

**Published:** 2020-12-16

**Authors:** Dinesh Kumar Kandaswamy, Makarla Venkata Sathya Prakash, Jochen Graw, Samuel Koller, István Magyar, Amit Tiwari, Wolfgang Berger, Sathiyaveedu Thyagarajan Santhiya

**Affiliations:** 1 Department of Genetics, Dr. ALM Post Graduate Institute of Basic Medical Sciences, University of Madras, Tamil Nadu 600 113, India; v_santhiya63@hotmail.com; 2 Institute of Developmental Genetics, Helmholtz Zentrum Munchen, D-85764 Munich, Germany; 3 School of Optometry and Vision Sciences, Cardiff University, Cardiff CF24 4HQ, UK; 4 Regional Institute of Ophthalmology, Government Eye Hospital, Egmore, Chennai 600 008, India; drmvsp@gmail.com; 5 Institute of Medical Molecular Genetics, University of Zurich, 8952 Schlieren, Switzerland; koller@medmolgen.uzh.ch (S.K.); istvan.magyar@team-w.ch (I.M.); amittiwari.ethz@gmail.com (A.T.); berger@medmolgen.uzh.ch (W.B.); 6 Zurich Center for Integrative Human Physiology, University of Zurich, 8057 Zurich, Switzerland; 7 Neuroscience Center Zurich, University and ETH Zurich, 8057 Zurich, Switzerland

**Keywords:** congenital cataract, WES, FYCO1, PAX6, EPHA2, P3H2, TDRD7, NCOA6, hearing and speech impairment, genetic heterogeneity, clinical heterogeneity

## Abstract

Congenital cataracts are the prime cause for irreversible blindness in children. The global incidence of congenital cataract is 2.2–13.6 per 10,000 births, with the highest prevalence in Asia. Nearly half of the congenital cataracts are of familial nature, with a predominant autosomal dominant pattern of inheritance. Over 38 of the 45 mapped loci for isolated congenital or infantile cataracts have been associated with a mutation in a specific gene. The clinical and genetic heterogeneity of congenital cataracts makes the molecular diagnosis a bit of a complicated task. Hence, whole exome sequencing (WES) was utilized to concurrently screen all known cataract genes and to examine novel candidate factors for a disease-causing mutation in probands from 11 pedigrees affected with familial congenital cataracts. Analysis of the WES data for known cataract genes identified causative mutations in six pedigrees (55%) in *PAX6, FYCO1* (two variants)*, EPHA2*, *P3H2,* *TDRD7* and an additional likely causative mutation in a novel gene *NCOA6*, which represents the first dominant mutation in this gene. This study identifies a novel cataract gene not yet linked to human disease. NCOA6 is a transcriptional coactivator that interacts with nuclear hormone receptors to enhance their transcriptional activator function.

## 1. Introduction

Congenital cataracts (CC) are the leading cause of significant vision loss, accounting for one-third of childhood blindness globally. The global incidence of congenital cataracts ranges from 2.2–13.6 per 10,000 children, with the highest prevalence being estimated in Asia (7.43/10,000) [1]. While 50% are of the hereditary nature is autosomal-dominant with complete penetrance, autosomal recessive or X-linked types are rare. Cataracts can lead to permanent blindness by interfering with the sharp focus of light on the retina, ensuing in a failure to establish appropriate visual cortical synaptic connections with the retina. Despite the advancement in surgical management and efficient visual rehabilitation, prompt early diagnosis is important to ensure visual outcome in cataract surgeries to prevent blindness. Classical linkage and candidate gene analyses have established the underlying genetic heterogeneity in congenital cataracts, which reports at least 38 associated genes and 45 mapped loci so far [2]. However, there are more than 100 genes that are known to be causative of either syndromic or non-syndromic CCs with intrafamilial and interfamilial variability as reported in the Catmap database (http://cat-map.wustl.edu/). In a recent review on guide to suspect the genetic basis of cataractogenesis, the authors have categorized the genes based on the phenotypes reported in the literature as syndromic cataract (104 genes), genes implicated in syndromes but with reports of only congenital cataracts (8 genes), only cataract causing genes (non-syndromic) (34 genes) and genes that are known to cause cataract along with other eye anomalies (11 genes) [3]. Such classification aids in the analysis of molecular basis of congenital cataract as it gives a clue to sort out the genes based on phenotypes and such classifications are helpful only if complete clinical documentation of the patients is known. The consequence of an untreated congenital cataract leads to an irreversible neurophysiological changes and sensory deprivation amblyopia with associated outcomes of strabismus and nystagmus [4]. It is well known that the best outcome is documented on congenital cataract surgery and optical corrections done before 6-8 weeks of age, thus emphasizing good ophthalmic examination combined with multi-disciplinary team work to achieve the best possible visual outcomes [4,5]. Early diagnosis is often a daunting task due to the numerous differences in an infant eye compared to an adult. And often children with cataract are microophthalmic with hypoplastic and vascular iris and may exhibit microcoria with immature trabecular meshwork and a shallow anterior chamber with lack of rigid sclera [6]. Thus, prompt diagnosis of congenital cataract and knowing its molecular basis not only enables to achieve optimum visual outcome but aids in further management of the secondary disorders caused during the treatment for the primary disease [4]. The conventional candidate gene screening approach offers a limited scope for genetic diagnosis in clinical practice. Hence, whole-exome sequencing (WES) has recently been successfully applied in mapping disease-causing genes and mutations for Mendelian diseases and tumors. Employing exome capture chips followed by next-generation sequencing comprehensively explores the potential structural or functional variants in the exome. This study therefore aims to establish the molecular basis of congenital cataracts in registered families (*n* = 11) through the whole-exome sequencing technique. The implementation of next-generation sequencing (NGS) technologies in diagnostic genetic testing has led to the rapid identification of pathogenic mutations in familial cases of inherited CCs. In this study, we adopted whole-exome sequencing (WES) in probands of 11 cohorts representing clinically diverse phenotypes of congenital cataracts. We could identify putative disease-causing variants that enable a genetic diagnosis in 55% of the cases. Further, it is noted that autosomal recessive forms (9/11) of cataracts do exist in South India in view of the social practice of consanguineous marriages. The identified mutation spectrum included novel variants of already known candidates of CCs and a novel potential candidate gene. Unresolved cases might be instrumental towards the identification of novel disease-associated genes.

## 2. Results

### 2.1. Clinical Documentation and Pattern of Inheritance

In the present study, we analyzed 11 families for whole-exome sequencing based on sample availability. Six families were resolved, marking the success rate as 55%. The complete clinical documentation of the resolved six families are as detailed in Table 1. An autosomal-recessive pattern of inheritance is observed in the majority of the recruited families, followed by an autosomal-dominant pattern of inheritance. Most of the probands and affected family members had a visual acuity of either 6/60 or less (Table 1).

### 2.2. Gene Mutation Identification From Whole-Exome Sequencing and Validation Through Sanger Sequencing

DNA samples of 11 probands were sequenced with NextSeq500 with an average of 39,000 variants obtained through WES. The mean coverage was 200× with 88% of reads > 30×. Annotation of the raw seqeunce data, variant calling, filtering and analysis were done as described in the methods. The whole-exome data were sorted and filtered based on biological context and pathogenicity and homozygosity/heterozygosity (based on the pattern of inheritance in each family), which led to the identification of a novel mutation in known cataract genes, as well as some novel genes not yet associated with congenital cataracts (Table 2). The mutations were analyzed using different in silico prediction tools. Missense mutations were analyzed using Proven, SIFT, POLYPHEN-2, Mutation Accessor, Panther and I-Mutant, while other mutations, including missense, were also analyzed using Mutation taster, PhyloP and PhastCons, scores to show the conserved nature of the nucleotide across species. The scores of individual variations identified in this study are validated and classified according to ACMG criteria as detailed in Table 2.

### 2.3. Congenital Cataract Families Resolved for Genetic Mutations Using WES

Initial candidate gene screening of eight selected genes (based on the frequency of mutation reported in the literature) in 11 congenital cataract probands did not reveal any mutations, but only synonymous sequence variations (SNPs) were documented. The subsequent adoption of next-generation sequencing facilitated a successful molecular dissection in 55% of the families investigated with congenital cataracts. Out of 11 families studied, five cases were shown to have novel putative disease-causing variants and one with an already known mutation. This study also documented mutations in a novel gene, *NCOA6*, to be a putative gene for syndromial congenital cataracts and associated hearing and speech impairments. The mutations documented in cases with congenital cataracts are summarized as follows.

### 2.4. Family Code: DKEC4

**Figure 1:**
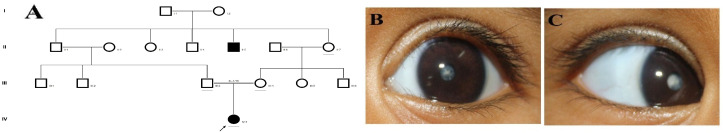

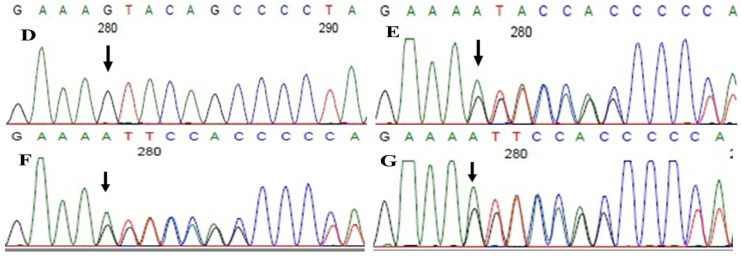

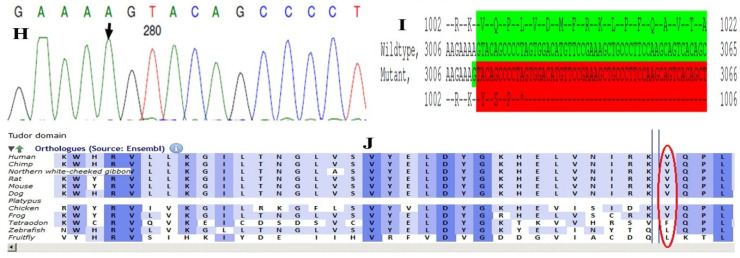
The pedigree of DKEC4 (**A**). Eye picture of the proband’s (IV:1) right eye (**B**) and left eye (**C**) depicting posterior subcapsular cataracts. Partial sequence chromatogram of the DKEC4 family members, *TDRD7* exon 16, depicting a homozygous mutation (c.3012 del; p.Val1005Tyrfs*4) in the proband ((**D**)—IV:1). Other family members: mother ((**E**)—III:4), father ((**F**)—III:3) and grandmother ((**G**)—II:7) were heterozygous for this variant control subject (**H**), showing a wildtype sequence. Site of mutation marked by an arrow. Mutational consequence leading to frame shift (in red) compared with wildtype sequence (in green) (**I**). Amino acid sequence alignment of human TDRD7 and orthologs from other species, showing a conservation of p.V10005 (encircled in red) (**J**). Divergent amino acid residues are shaded in a white color background.

Family history revealed an autosomal recessive mode of inheritance (Figure 1A), as both the parents were unaffected, and there was one affected member in the second generation, a distant relative (grandfather’s brother) of the proband, with similar congenital cataracts and was also reported to be infertile by the grandmother. The proband depicted posterior polar cataracts (Figure 1B,C), and other abnormalities were detected upon either an ophthalmologic or general physical examination (specifically, ocular movements were full, conjunctivae were clear and the anterior chambers looked normal). The WES analysis identified a novel homozygous mutation in the gene *TDRD7,* the deletion of the last nucleotide A of codon 1004 (AAA, coding for lysine) that resulted in a frame shift mutation, which subsequently introduced a premature stop codon in exon #16 (c.3012 del; p.Val1005Tyrfs*4) (Figure 1D,I). This is a novel mutation that probably causes autosomal recessive cataracts in this family. Bidirectional sequencing of exon 16 of the *TDRD7* gene of the proband (IV.1) confirmed the homozygous deletion at position c.3012. This deletion neither creates nor deletes any restriction site; hence, all the available family members were checked through PCR-based sequencing of exon 16 of the gene *TDRD7*, and all the unaffected family members were heterozygous carriers of the deletion (Figure 1E–G). Insilico analysis using varsome shows this null variant (frame-shift), in gene TDRD7, for which loss-of-function is a known mechanism of disease (gene has 6 pathogenic LOF variants and LOF *Z*-Score = 4.41 is greater than 0.7), associated with Cataract, thus this variant is categorized as pathogenic based on ACMG criteria on the following conditions, PVS1 (null variant), PM2 (variant not found in gnomAD exomes and genomes), PP3 (pathogenic computational predictions). This mutation was also cross-checked in unrelated control subjects (*n* = 100) of the same ethnicity, screened through PCR-based direct sequencing and found to be absent (data not shown).

### 2.5. Family Code: C343

**Figure 2:**
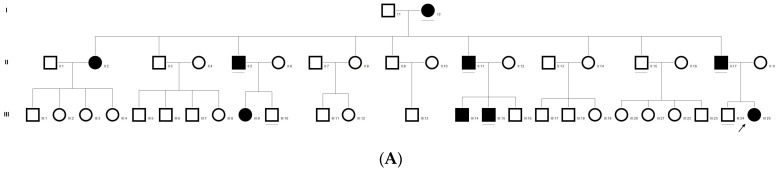

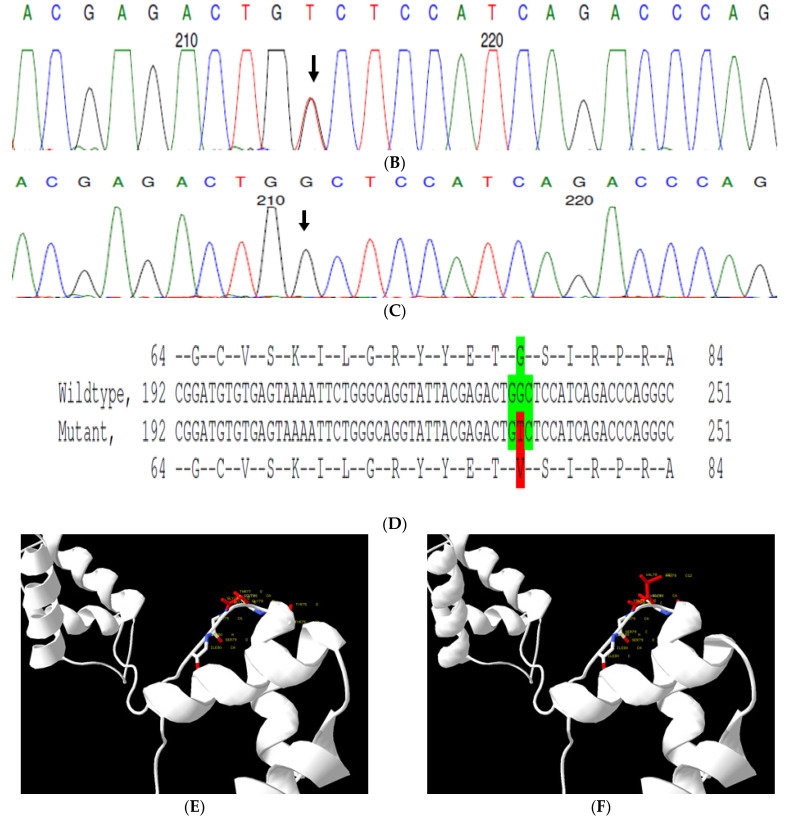

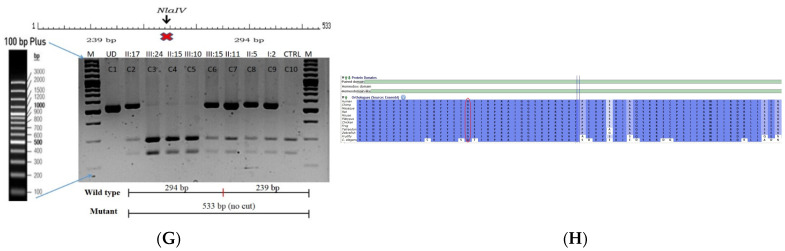
Pedigree of C343 (**A**). Partial sequence chromatogram of C343 family members, *PAX6* exon 7, depicting a heterozygous mutation (c.233 G > T; p.G78V) in the proband’s father ((**B**), II:17) and control subject (**C**). Mutant and wildtype peaks “T” and “G” are marked by an arrow. Missense mutation (in red) compared with the wildtype sequence (in green) (**D**). Model structure representing wildtype (**E**) versus mutant (**F**) PAX6. The mutated amino acid is highlighted in red, while its interacting amino acids nearby are marked in a white and blue combination. The mutated amino acid hinders the exposure of neighboring residues such as threonine at position 77 and tyrosine at position 75 and, also, alters other interactions, which might affect its interaction with its downstream targets. RFLP analysis of the mutation p.G78V in *PAX6* (**G**), which shows co-segregation of the heterozygous mutation among affected family members (C2 and C6-C9). Unaffected family members (C3-C5) and control (DKC192) (C10) show complete digestion. C1—undigested PCR product. Amino acid sequence alignment of human PAX6 and orthologs from other species (**H**), showing the conservation of p.G78 (encircled in red). Divergent amino acid residues are shaded in a white background.

This is a retrospective case documented to have anterior polar cataracts with microcornea. Family history revealed an autosomal dominant mode of inheritance (Figure 2A); multiple relatives of the probands with similar phenotypes were documented. The WES analysis led to the identification of a G > T transversion in exon #7 of the *PAX6* gene, resulting in a missense mutation (c.233 G > T; p.Gly78Val) (Figure 2B–D). This mutation deletes a restriction site for the enzyme, *NlaIV*; a Restriction Fragment Length Polymorphism (RFLP) analysis among available family members indicated segregation of the mutation among the affected family members (Figure 2G) but its absence in 120 ethnically matched control subjects (data not shown). Both the nucleotide (which is evident by PhyloP (score 6.014) and PhastCons (score 1)) and the amino acid (which is evident by a high score in PANTHER—1037) are highly conserved across species (considering 12 species, up to *Caenorhabditis elegans*) (Figure 2H). In-silico tools predict this variation to be pathogenic (Provean, −7.35; SIFT, 0; POLYPHEN2, 1; mutation assessor, 3.78 and mutation taster, 1) (Grantham dist: 109 (0–215)). A Mutpred analysis also revealed it to be a deleterious mutation, with a probability of 0.951, loss of disorder (*p* = 0.0529), gain of molecular recognition features (MoRF) binding (*p* = 0.0751), loss of glycosylation at S79 (*p* = 0.1159), loss of phosphorylation at S79 (*p* = 0.1323) and loss of catalytic residue at T77 (*p* = 0.1757). Homology modeling of the mutant and wildtype proteins revealed that mutated residue alters the exposure of neighboring amino acids, which might affect the protein interaction with its downstream targets (Figure 2E,F). Varsome analysis revealed that this variant as likely Pathogenic based on ACMG criteria—Hot-spot of length 51 base-pairs has 14 non-VUS missense/in-frame variants (14 pathogenic and 0 benign), pathogenicity = 100.0%, qualifies as hot-spot. Furthermore, 187 out of 199 non-VUS missense variants in gene PAX6 are pathogenic = 94.0% which is more than threshold of 51.0%, and 418 out of 507 clinically reported variants in gene PAX6 are pathogenic = 82.4% which is more than threshold of 12.0%. Clinvar also categorizes this variant as pathogenic based on previous reports. Thus this variant is categorized as pathogenic based on ACMG criteria on PM1 (hot spot of disease causing variations), PM2 (variant not found in gnomAD exomes and genomes) and PP5 (based on clinvar classification as pathogenic based on two submissions).

### 2.6. Family Code: CCE13

**Figure 3:**
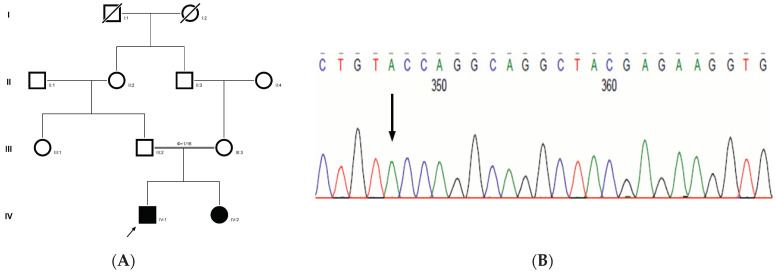

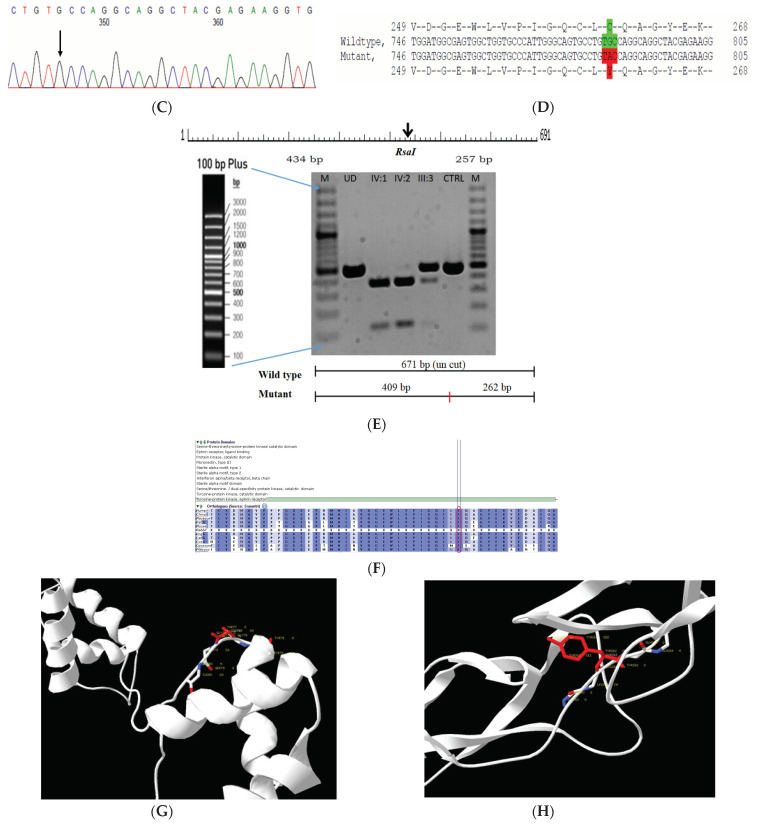
Pedigree of CCE13 (**A**). Partial sequence chromatogram of the proband ((**B**), IV:1) and control subject (**C**), *EPHA2* exon 3, depicting a homozygous mutation (c.785G > A; p.Cys262Tyr). Mutant and wildtype nucleotide peaks “A” and “G” marked by an arrow. Missense mutation (red) compared with wildtype sequence (green) (**D**). RFLP analysis of the mutation p.C262Y in *EPHA2* (**E**), which shows co-segregation of the homozygous mutation among affected family members (IV:1 and IV:2). Unaffected mother as the carrier (III:3). Control (DKC192) (CTRL) shows no digestion. UD—undigested PCR Product. Amino acid sequence alignment of human EPHA2 and orthologs from other species (**F**), showing conservation of p.C262 (encircled in red). Divergent amino acid residues are shaded in a white background. Model structure representing wildtype (**G**) and mutant (**H**) EPHA2. The mutated amino acid site is marked in red, while its interacting amino acids at the neighborhood are marked in a white and blue combination. The large physiochemical difference between cysteine and tyrosine alters its interactions with neighboring amino acids, which changes the structural confirmation of the protein. Secondary structure alterations were seen.

The proband had bilateral lamellar cataracts with microcornea and nystagmus with an autosomal-recessive pattern of inheritance (Figure 3A). He had an affected sister with similar complaints of nystagmus, who, upon clinical investigations, revealed lamellar cataracts with microcornea. A G > A transition was observed in exon #3 of *EPHA2*, resulting in a novel missense mutation (TAC) (c.785G > A; p.Cys262Tyr) (Figure 3B–D) that creates a restriction site for the enzyme *RsaI*. A RFLP analysis performed among family members revealed the affected sibling as homozygous, while the mother was a carrier of this mutation (Figure 3E) and was found to segregate among the affected family members. Both the amino acid residue and the nucleotide are highly conserved across species (considering 11 species up to *Platypus*) (which is evident by PhyloP (score 6.014) and PhastCons (score 1)) (Figure 3F). In-silico tools predict this variation to be pathogenic (PROVEAN, −8.84; SIFT, 0; POLYPHEN2, 0.658; mutation assessor, 3.755; and mutation taster, 1) (Grantham dist: 194 (0–215)). Homology modeling of the wildtype and mutant proteins depict alterations in the secondary structure leading to change in its overall structure, which might affect its function (Figure 3G,H). This variant is categorized as variant of uncertain significance based on ACMG criteria on PM2 (variant not found in gnomAD exomes and genomes) and PP3 (pathogenic computational verdict based on 12 pathogenic predictions) as well as BP1 supporting (29 out of 40 non-VUS missense variants in gene EPHA2 are benign = 72.5% which is more than threshold of 51.0%, and 75 out of 135 clinically reported variants in gene EPHA2 are benign = 55.6% which is more than threshold of 24.0%.).

### 2.7. Family Code: CCE27

**Figure 4:**
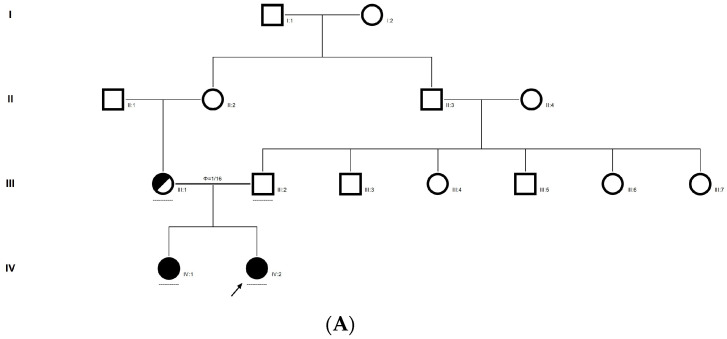

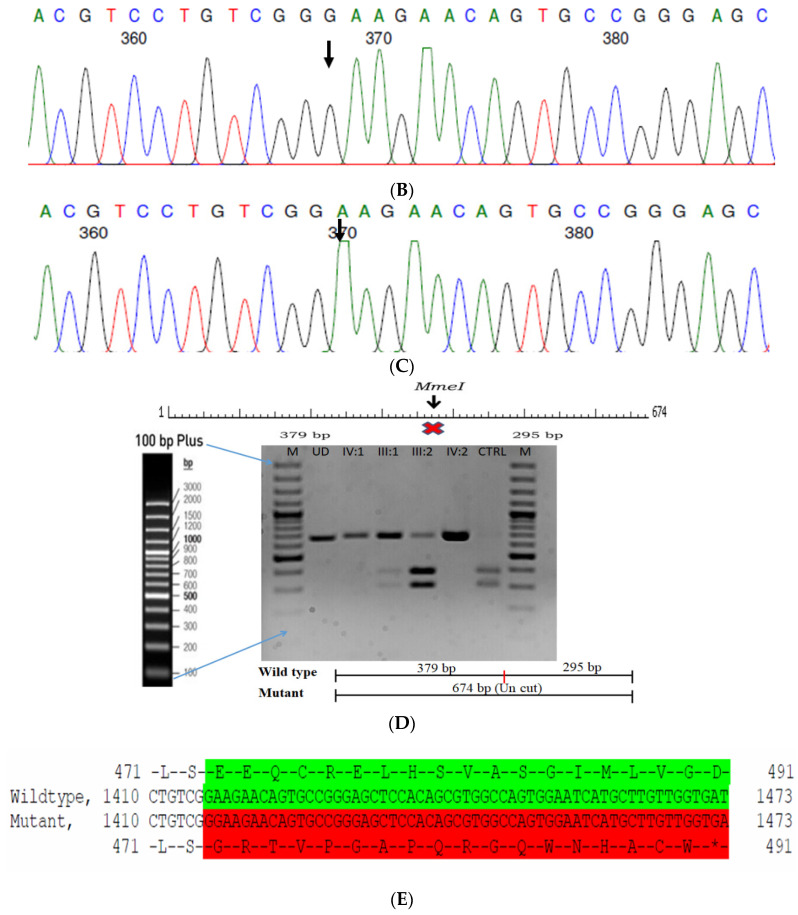
Pedigree of CCE27 (**A**). Partial sequence chromatogram of the proband ((**B**), IV:1) and control subject (**C**), *P3H2* exon 9, depicting a homozygous mutation (c.1417 dup; p.Glu473Glyfs*19). Mutant and wildtype peaks are marked by an arrow. RFLP analysis of the mutation p.Glu473Glyfs*19 in *P3H2* (**D**), which shows co-segregation among the affected family members (IV:1 and IV:2), while the parents are carriers (III:1 and III:2). Control (DKC192) (CTRL) shows complete digestion. UD—undigested PCR Product. The variation leading to frame shift mutation (red) compared with the wildtype sequence (green) (**E**).

The proband had bilateral total patchy cataract of the autosomal recessive pattern of inheritance type (Figure 4A). The proband had an affected sister with similar complaints, who, upon clinical investigation, revealed total cataracts. The mother had a milder cataract phenotype expressed in her late-teen ages. A novel homozygous mutation in the gene *P3H2* (duplication of nucleotide G) that affects the second nucleotide of codon #473, GAA (glutamine), resulted in a frame-shift mutation, which, subsequently, introduced a premature stop codon in exon #9 (c.1417 dup; p.Glu473Glyfs*19) (Figure 4B–E). This is a novel mutation identified to cause congenital cataracts that segregates among the affected family members. The proband’s mother, being a carrier, had a milder phenotype (probably due to haplo insufficiency) compared to her affected daughters. This mutation deletes a restriction site for the enzyme *MmeI*. The RFLP analysis of this mutation among the family members revealed that the affected sibling is homozygous, while both the mother and father were carriers for this mutation (Figure 4D). This duplicated nucleotide is highly conserved across species (which is evident by PhyloP (score 3.016) and PhastCons (score 1)). In-silico tools predict this variation to be pathogenic (mutation taster, 1). About one-third of the amino acid residues are lost, which includes the loss of several sites, such as the Fe2OG dioxygenase domain (557–671): 705–708, a motif that prevents secretion from the endoplasmic reticulum (ER); an active site of the enzyme 662; a carbohydrate attachment site 549 (*N*-linked GlcNac) and iron-binding sites (580, 582 and 652). Hence, the mutated protein is nonfunctional; however, there are possibilities of a nonsense-mediated decay of the transcript as well. This variant is categorized as pathogenic based on ACMG criteria on PVS1 (Null variant (frame-shift), in gene P3H2, for which loss-of-function is a known mechanism of disease (gene has 11 pathogenic LOF variants and LOF *Z*-Score = 0.771 is greater than 0.7), associated with Myopia, high, with cataract and vitreoretinal degeneration.) and PM2 (variant not found in gnomAD exomes and genomes) as well as PP3 (Pathogenic computational predictions).

### 2.8. Family Code: ACR12

**Figure 5:**
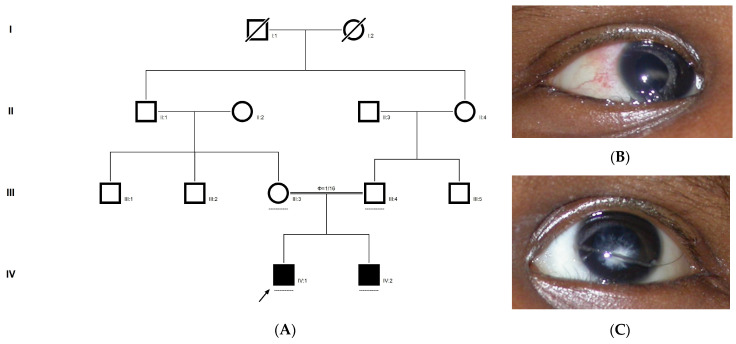

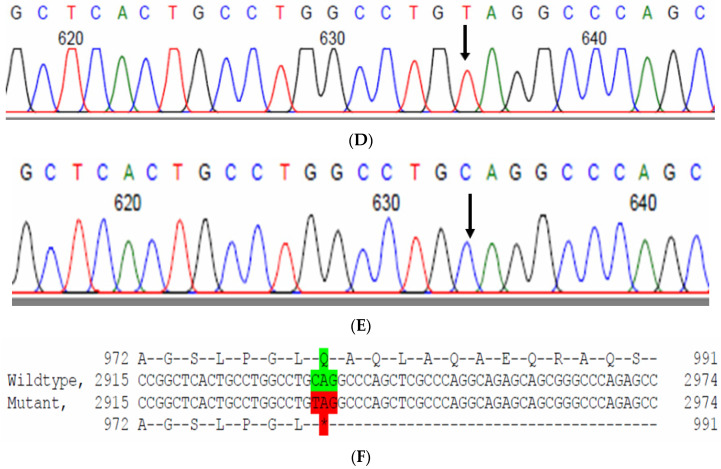

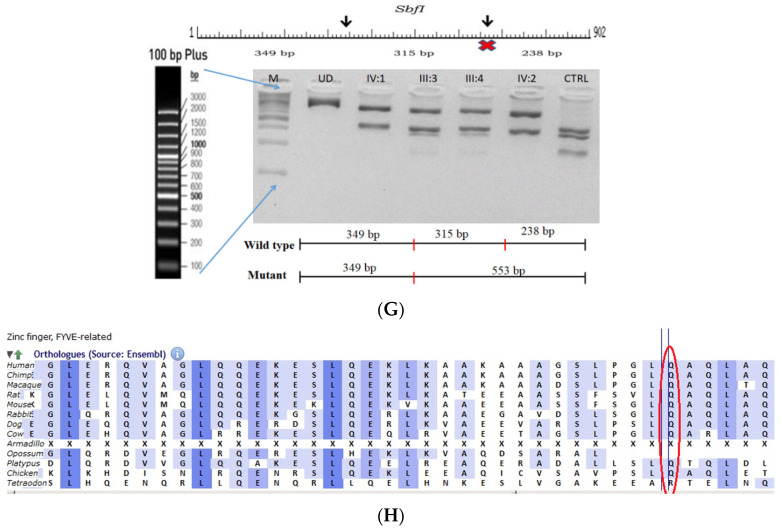
Pedigree of ACR12 (**A**). Eye picture of the proband (IV:1)—right eye (**B**) depicting posterior cortical cataracts and left eye (**C**) depicting Lamellar cataracts. Partial sequence chromatograms of the proband ((**D**), IV:1) and control subject (**E**), *FYCO1* exon 8, depicting a homozygous mutation (c.2935C > T; p.Gln979*). Mutant and wildtype nucleotide peaks “T” and “C” marked by an arrow. Mutational consequence leading to truncation (in red) compared with the wildtype sequence (in green) (**F**). RFLP analysis of the mutation p.Gln979* in *FYCO1* (**G**), which shows co-segregation among the affected family members (IV:1 and IV:2). Unaffected parents are carriers (III:3 and III:4). Control (CTRL-DKC190) shows complete digestion. UD—undigested PCR product of the control sample. Amino acid sequence alignment of human FYCO1 and orthologs from other species (**H**) showing the conservation of p.Q979 (encircled in red).

The proband had posterior cortical cataracts in his left eye (LE) and lamellar cataracts in his right eye (RE) (Figure 5B,C), with an autosomal-recessive pattern of inheritance (Figure 5A). He had an affected sister with similar complaints, who, upon clinical investigation, revealed a cataractous phenotype. A novel homozygous nonsense mutation, viz., c.2935C > T; p.Gln979* in the *FYCO1* gene, was observed (Figure 5D–F). This mutation deletes a restriction site for the enzyme, *Sbf1*; co-segregation of the same was confirmed by Sanger sequencing, as well as through RFLP. This novel truncation mutation was found to co-seggregate among the affected individuals (Figure 5G). The mutated nucleotide is highly conserved across species (which is evident by PhyloP (score 5.648) and PhastCons (score 1)) (Figure 5H). The novel mutation, p.Gln979*, resides in the coiled coil domain of *FYCO1*, leading to a loss of three critical domains (FYVE and GOLD), including the coiled domain of the protein. Hence, this mutation is predicted to interfere with the intracellular transport of autophagocytic vesicles from the perinuclear area to the periphery, thus leading to the accumulation of a large number of vesicles and, thereby, causes a loss of lens transparency. In-silico tools predict this mutation to be pathogenic (PROVEAN, −7.15 and mutation taster, 0.99). This variant is categorized as pathogenic according to ACMG criteria based on PVS1 (Null variant (nonsense), in gene FYCO1, for which loss-of-function is a known mechanism of disease (gene has 11 pathogenic LOF variants and LOF *Z*-Score = 2.17 is greater than 0.7), associated with Cataract, autosomal recessive congenital 2.), PM2 (variant not found in gnomAD exomes and genomes) and PP3 (pathogenic computational verdict based on 5 pathogenic predictions).

### 2.9. Family Code: BCC23

**Figure 6:**
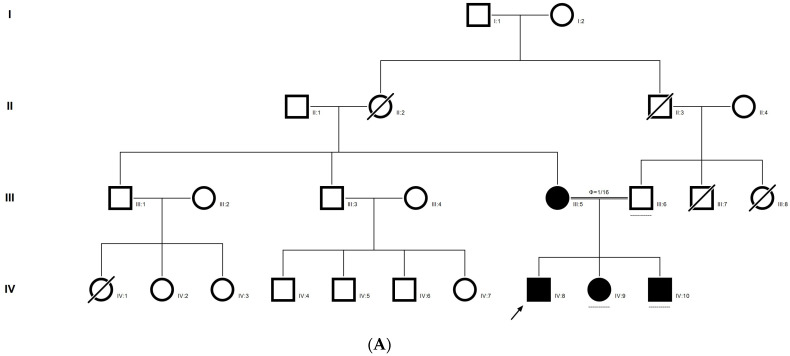

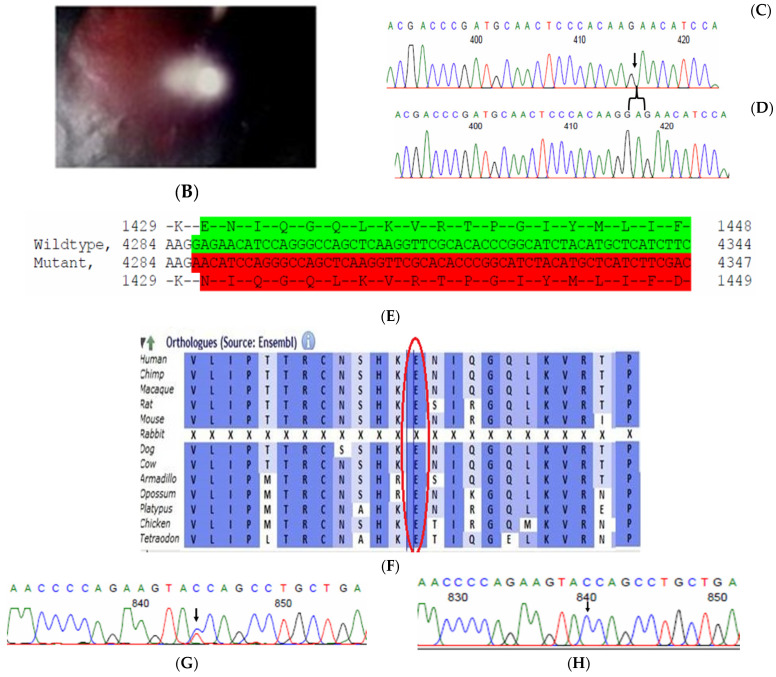

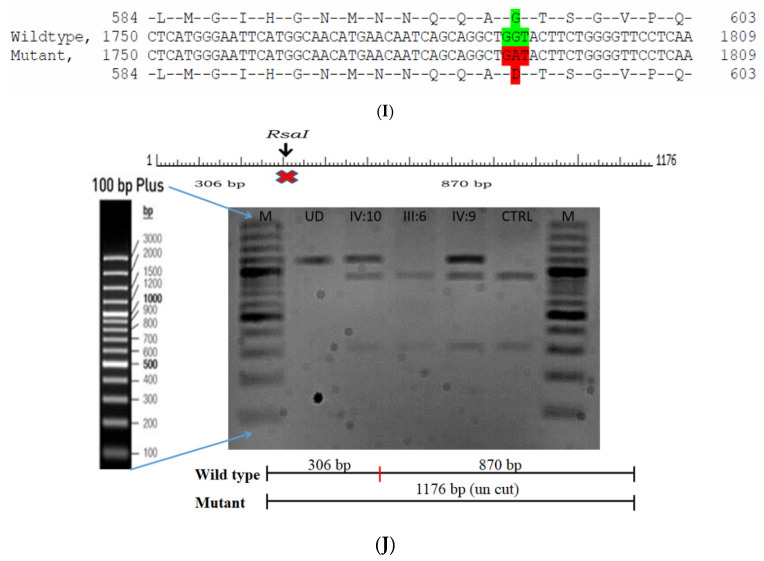

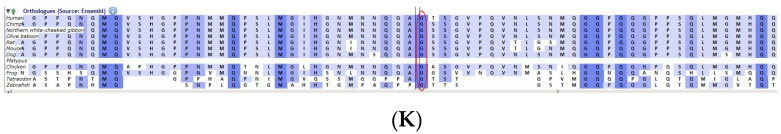
Pedigree of BCC23 (**A**). Clinical phenotype of probands (IV:8): right eye depicting posterior subcapsular cataracts (**B**) upon retro-illumination. Partial sequence chromatogram of BCC23 family members, *FYCO1* exon 17, depicting a homozygous mutation (c.4288_4290del; ΔE1430) in the proband’s brother (IV:10) (**C**), and control subject (**D**) showing a wildtype sequence. Site of mutation was marked by an arrow. The mutation leads to an in-frame deletion of a single amino acid (marked in red) compared with the wildtype sequence (marked in green) (**E**). Amino acid sequence alignment of human FYCO1 and orthologs from other species (**F**), showing a conservation of p.E1430 (encircled in red). Partial sequence chromatograms (reverse) of the proband’s affected brother ((**G**), IV:10) and control subject (**H**), *NCOA6* exon 9, depicting a homozygous mutation (c.1790G > A; p.Gly597Asp). Mutant and wildtype peaks “T” and “C” marked by an arrow. Missense mutation (marked in red) compared with the wildtype sequence (marked in green) (**I**). RFLP analysis (**J**) of the variant allele p.Gly597Asp in *NCOA6*, which shows co-segregation among the affected family members (IV:9 and IV:10). Unaffected father (III:6) and control (CTRL-DKC190) shows complete digestion, indicating a wildtype sequence. UD—undigested PCR product. Amino acid sequence alignment of human NCOA6 and orthologs from other species, showing a conservation of p.G596 (encircled in red). Divergent amino acid residues are shaded in a white background (**K**).

The proband who is deaf and dumb had dense cortical cataracts in his LE and posterior subcapsular cataracts in his RE (Figure 6B). He had two affected siblings, who had posterior subcapsular cataracts, depicting an autosomal-recessive pattern of inheritance (Figure 6A), and both siblings were deaf and dumb. The mother had mild blue dot opacities in both her eyes, while the father was unaffected. Two possible variants were documented in WES to be causative in this family. One of the variants occurred in the gene *FYCO1* as an in-frame deletion of three base pairs, leading to a loss of a single amino acid (c.4288_4290del; p.Glu1430del) (Figure 6C–E). This variant was homozygous in the two affected siblings, and the unaffected proband’s father was a heterozygous carrier for this variant (data not shown). The deleted amino acid residue is highly conserved across species (Figure 6F). The deleted three nucleotides were conserved across species (PhyloP (scores 1.5, 4.301 and 5.18 values for individual nucleotides in the order of deletion) and PhastCons (score 1 for all the three nucleotides)). This variant was observed in the Gnom AD exome database (Formerly known as Exome Aggregation Consortium (EXAC) database) in the Southern Indian population at a very low frequency (0.00002) (no homozygotes observed) (Supplementary Table S1). This variant is categorized as variant of uncertain significance based on ACMG criteria considering PM2 (GnomAD exomes homozygous allele count = 0 is less than 3 threshold for recessive gene *FYCO1* (good gnomAD exomes coverage = 96.6).Variant not found in gnomAD genomes (good gnomAD genomes coverage = 31.3)) and PM4 (inframe variant in gene *FYCO1* which is not in the repeat regions) as well as PP3 supporting (pathogenic computational prediction).

A heterozygous variant in the gene *NCOA6* leading to a missense mutation (c.1790G > A; p.Gly597Asp) was observed in the proband’s sample (Figure 6G–I). This mutation deletes a restriction site for the enzyme *RsaI.* A RFLP analysis revealed that both the affected siblings with similar phenotypes (both were deaf and dumb) had this heterozygous variant but not in the unaffected father (Figure 6J). We could not analyze it in the mother, as it was a retrospective case. In-silico tools predicted the *NCOA6* variation to be pathogenic (PROVEAN, −1.52; SIFT, 0.008; POLYPHEN2, 0.949; mutation assessor, 1.955 and mutation taster, 1). The mutated nucleotide is highly conserved across species (which is evident by PhyloP (score 4.321), PhastCons (score 1) and Panther (which is evident by a high PANTHER score of 750) (Figure 6K). This variant was observed in the Genome Aggregation Database (gnomAD exomes) in the Southern Indian population at a very low frequency (0.00009) (no homozygotes observed) (Supplementary Table S2). Some of the in-silico tools predicted this variant to be deleterious and probably damaging. However, this variant is categorized as likely benign based on ACMG criteria on PM2 (GnomAD exomes allele count = 3 is less than 5 threshold for gene NCOA6 (good gnomAD exomes coverage = 60.5). Variant not found in gnomAD genomes (good gnomAD genomes coverage = 28.3), BP1 supporting (17 out of 17 non-VUS missense variants in gene NCOA6 are benign = 100.0% which is more than threshold of 51.0%, and 35 out of 35 clinically reported variants in gene NCOA6 are benign = 100.0% which is more than threshold of 24.0%.) and BP4 supporting (6 pathogenic prediction and 7 benign computational prediction). A mutpred analysis revealed that this had less probability for being deleterious (*p* = 0.207), whereas the same tool predicted that this mutation might cause a loss of MoRF binding (*p* = 0.0575), loss of catalytic residue at A596 (*p* = 0.1278), gain of solvent accessibility (*p* = 0.1583), loss of glycosylation at S599 (*p* = 0.2023) and gain of relative solvent accessibility (*p* = 0.2363). The FYCO1 variant (c.4288_4290del; p.Glu1430del) is also predicted to be pathogenic (PROVEAN, −7.62 and mutation taster, 0.99), with the loss of the critical domain GOLD. However based on previous studies, the NCOA6 variant could act as a modifier along with the FYCO1 variant and could be the plausible cause of the syndromic cataract reported in the family.

## 3. Discussion

Congenital/pediatric cataracts is the leading cause of treatable childhood blindness globally. Congenital cataract affects the quality of vision, as it hinders the transmission of light to the retina during the critical period of visual development, which may lead to irreversible blindness. Earlier studies have reported the difficulties in the prognosis/early diagnosis of congenital cataract, as this is crucial in achieving better treatment outcome, A recent study has shown that only fewer cases (less than 50%) requiring cataract surgery under the age of 3 years of age were referred before 9 weeks of age, and emphasizes early diagnosis requires skilled professionals for a definite diagnosis [7]. This prompts the necessity for early diagnosis and timely clinical intervention to avoid blindness or severe visual impairment. Recent meta-analysis and systematic reviews indicate that south Asia has the largest prevalence (7.43/10,000) of congenital cataract [1].

In the present study, a candidate gene screening panel consisting of eight genes in 11 families with congenital cataracts revealed no putative mutations in any of the families. The major limitation in this approach is that screening of all the reported 40 genes involved in cataractogenesis is not only time-consuming but laborious as well. Hence, alternative rapid approaches as either panel sequencing or whole-exome sequencing are being adopted as the method of choice to detect the causative mutations underlying cataracts. In this study, screening through whole-exome sequencing was adopted in 11 families short-listed based on the size of the kindred and the maximum number of available samples. Pedigrees registered through prospective (*n* = 6), as well as retrospective, studies (*n* = 5) were taken up for whole-exome sequencing. This method was promising towards characterizing the molecular basis in at least six out of the 11 families screened (already screened for eight genes), which is about 55 percent compared to the approach by candidate gene screening.

However, the limitations in our study includes some of the retrospective cases have limited samples and not extensive clinical documentations were available, which halted further analysis. Furthermore, there are always possibility of the role of non-coding genes and epigenetic mechanism which could play a crucial role in certain cases, where the causative mechanism is yet to be identified. A recent review on management of pediatric cataracts has reviewed the effect of early and late post-operative complications on the better visual outcome, suggesting recommendations on operational procedures in children less than 6 years and suggest hydrophobic foldable acrylic posterior chamber intraocular lens (PCIOL) to lower the post-operative inflammation. They have also suggested that timely detection aids in the proper long-term management addressing the post-operative visual rehabilitation such as refractive correction treatment of concomitant amblyopia and bifocal correction [8]. With the advancement in molecular diagnosis, clinical prognosis can be made appropriately and on time for better management of the disorder. In our study, one of the family (DKEC4), the family members were informed about the genetic condition and was suggested if any new born in their extended family could do the genetic test for an early prognosis and management of the disorder for a better visual outcome and they have enrolled for special tests. Recently one of the newborn in their extended family members was tested positive, and was under continuous management, which aid in the timely management of the disorder for a better outcome. Thus knowing the disease variant aids in the future management of the disease in the extended family members, especially like our cohort (similar to all Asian population), where in consanguineous marriages are still in practice leading to successive disease phenotypes in the future offspring’s.

The spectrum of mutations identified varied as two-frame shift mutations in *P3H2* and *TDRD7*; one nonsense mutation in *FYCO1* and three missense mutations in *NCOA6*, *PAX6* and *EPHA2*. One in-frame deletion was also detected in *FYCO1*. The scope of whole-exome sequencing lies in the possibility of the identification of novel genes; accordingly, in the present study, we documented a novel gene, *NCOA6*, with a missense variation that is likely to be the molecular lesion underlying the posterior capsular cataracts in the family BCC23; this variant is also associated with the deaf and dumb phenotype as well.

As per the literature, the success rate of mutation detection was enhanced by targeted panel sequencing and whole-exome sequencing. Employing targeted panel sequencing comprised of 34 genes, Sun et al. (2014) [9] identified putative disease-causing variants in 50% of the families screened (nine out of 18 families), while it was 70% (46 cases comprised of both sporadic and familial origins) by screening 32 cataracts-associated genes through panel sequencing [10]. Zhai et al. (2017) [11] performed a panel sequencing of 54 cataracts-associated genes in 27 Han Chinese congenital cataracts families and was successful in detecting mutations in 17 out of 27 families studied. Few of the recent studies also focused on the genetic basis of sporadic cases. Li et al. (2016) [12] identified ~26% putative disease-causing variants (19 out of 74 patients) in sporadic congenital cataracts of the Han Chinese population through the panel sequencing of 61 lens-related genes.

In the present study, a four-generation pedigree with the skipping of a cataracts phenotype was documented. Candidate gene screening of eight genes in this family did not reveal any putative disease-causing variants. Whole-exome sequencing of the proband revealed a homozygous frame shift mutation, viz., p.Val1005Tyrfs*4 in the gene *TDRD7*. An analysis of the family members revealed that the proband’s parents and grandmother were carriers of this mutation; however, the other affected family member documented in the pedigree was not available for the study. The same mutation is absent in 120 ethnically matched control subjects, as verified by PCR-based direct sequencing. The mutation leads to the loss of the Tudor domain, which is crucial for its function. However, the frameshift occurs in exon no. 16 before the last exon, so there are possibilities for a nonsense-mediated decay of the transcripts.

TDRD7 is a Tudor domain containing a RNA-binding protein, which forms part of the RNA granules found to regulate cytoskeletal dynamics, movement of the cytocentrum and regulation and transportation of mRNA. They are expressed in lens fiber cells and male germ cells and are known to regulate the post-transcriptional modification of critical genes involved in lens development and spermatogenesis [13,14]. RNA granules determine the fate of a mRNA and are present in the form of processing bodies (PB) or stress granules (SG) in lower eukaryotes [15]. Lens fiber cells are functionally analogous to neurons and germ cells like sperm in several aspects, such as elongated nature, transcriptionally inactive and maintaining polarity. Recent studies have indicated that lens fiber cells also possess RNA granule-mediated post-transcriptional regulatory functions for its specialized morphology [13]. The mutation observed in this study (p.Val1005Tyrfs*4) results in the loss of less than 10% of the protein, leading to a slightly truncated protein; one of the critical Tudor domains is lost due to this mutation, which makes it nonfunctional, thereby causing congenital cataracts in the proband. However, there are also possibilities for a nonsense-mediated decay of the mutant transcript.

In the present study, the family C343 was documented to have a mutation in *PAX6* (p.Gly78Val). About 472 unique *PAX6* variants have been reported to cause a spectrum of eye diseases and other anomalies (http://lsdb.hgu.mrc.ac.uk/home.php?select_db=PAX6). PAX6 mutations are known to cause mainly Aniridia; however, other ocular anomalies, including microcornea, microphthalmia, congenital cataracts, keratitis, ocular coloboma, Peter’s anomaly, Gillespie syndrome, morning glory disc anomaly, foveal hypoplasia and optic nerve hypoplasia, have also been reported. This mutation in *PAX6* (G64V) is already reported to cause congenital cataracts with foveal hypoplasia [16]. The mutation as either G78V or G64V based on the splice variant identified in the current study is in the paired domain of PAX6 and causes autosomal-dominant congenital cataracts with microcornea in the kindred C343. Two-thirds of the reported missense mutations occur in the paired domain and may thus alter the binding specificity of some of the PAX6 targets [17]. Functional characterization of the paired domain missense mutation reported variations in either DNA-binding activity or transactivation activity of mutant PAX6 [18,19,20]. In general, the mutation in *PAX6* disturbs the entire downstream signaling cascade, leading to a spectrum of ocular phenotypes, as evidently documented in the literature, in several families with unusual phenotypes, along with cataracts.

The family CCE13, a four-generation pedigree (retrospective case) with affected offspring documented only in the fourth generation. Whole-exome sequencing analysis identified a novel putative disease-causing variant in the gene *EPHA2*, p.Cys262Tyr, which co-segregated among the affected individuals (IV:1 and IV:2) but was absent in the unaffected mother and 120 ethnically matched control subjects. Precisely, to date, the *EPHA2* mutation observed (C262Y) in the present study is the first one to be documented in the cysteine-rich region. Homology modeling of wildtype and mutant proteins revealed that the altered amino acid, tyrosine, affects the interactions with neighboring amino acids, leading to conformational changes in the protein, making it dysfunctional. As per the literature, only one family with a *EPHA2* mutation has been documented to have persistent fetal vasculature [21]. Persistent fetal vasculature (PFV) arises due to a failure in the regression of the hyaloid vasculature, which is one of the commonest causes of infantile cataracts. PFV is sometimes associated with microcornea and might cause secondary glaucoma due to elevated IOP (intra-ocular pressure). In the present study, the affected members of the family CCE13 are reported to have microcornea and nystagmus, along with lamellar cataracts; however, for further investigations, the family is not retrievable. A follow-up clinical study might answer several questions that might arise due to the multifunctional role of EPHA2.

A retrospective case CCE27 of three-generation pedigree depicting intra-familial clinical heterogeneity was documented in our earlier study. The mother had mild developmental cataracts, while her two daughters had severe patchy and total cataracts since birth, and the father was documented to have normal vision. Initial candidate gene screening did not reveal any putative disease-causing mutations; when the proband’s sample was investigated through whole-exome sequencing, a novel frame shift mutation in the gene *P3H2*, p.Glu473Glyfs*19 was identified. *P3H2* (Prolyl 3-hydroxylase) plays a critical role in collagen chain assembly, stability and crosslinking by catalyzing the post-translational 3-hydroxylation of proline residues in collagen. Collagens are the most abundant protein that constitutes up to about 30% of the total protein mass in mammals [22]. The present study documented a frame shift mutation, p.Glu473Glyfs*19, which resulted in the loss of one-third of the amino acids, leading to a truncated protein. Some critical domains (Fe2OG dioxygenase domain), active sites, signaling motifs and metal-binding sites necessary for the proper function of the enzyme are eventually lost due to this mutation. This mutation is predicted to undergo nonsense-mediated decay, and hence, loss of function of this gene could cause the cataract phenotype in the family. However, in earlier reports, the heterozygous carriers were documented to be normal, while, in the present study, a milder phenotype was documented in the heterozygous mother and is subjective to further analysis.

Whole-exome sequencing analysis of the proband (ACR12) revealed a pathogenic mutation in *FYCO1*, viz., p.Gln979*, which co-segregated with the affected individuals and was absent in 120 ethnically matched control subjects. As this mutation causes a premature stop codon, it might lead to nonsense-mediated decay of the transcript. FYCO1 is a phosphatidylinositol-3-phosphate-binding protein that functions like an adaptor, linking autophagosomes to microtubule plus-end-directed molecular motors. FYCO1 consists of a central long-coiled coil region with a helical RUN domain at its *N*-terminal, a FYVE domain, a LC3-interacting region and a GOLD domain at its *C*-terminal [23]. *Fyco1* expression in a mouse starts at E8.5, which remains stable until E12.5, then doubles until E16.5 to P12, with an eventual decrease in expression during the early embryonic stages [24]. From their observation, it is clear that the *FYCO1* expression is high during secondary fiber cell differentiation, which involves a denucleation process, thus substantiating its role in autophagy by clearing organelles. The degradation of aggregated misfolded proteins through autophagy is essential for regulating light scattering and lens transparency; any impairment in this process will lead to cataractogenesis. On another retrospective case, BCC23, four-generation kindred presented with a milder cataract phenotype as blue dot cataracts in the mother, whose children had severe cataracts phenotype, besides also being deaf and dumb. This family depicted intrafamilial phenotypic variability even within the same individual; the affected child’s LE had a dense cortical cataract, while the RE showed a posterior subcapsular cataract. Whole-exome sequencing of the proband revealed an in-frame deletion of a single amino acid in the *FYCO1* gene (p.Glu1430del). This variant has been reported in the ExAC browser in the South Asian population with a low allele frequency of 0.00024 with no homozygotes reported. This mutation lies in the GOLD domain, which might interfere with its function. However, the role of this variant in the pathogenesis of cataracts is unknown and needs to be functionally analyzed to resolve its direct effect on the protein function or its indirect action as a modifier. Two affected individuals, the proband’s brother and sister, were homozygous for this variant, while their father was a heterozygous carrier.

A novel variant in the gene *NCOA6* (p.Gly597Asp), hitherto not known to be linked with human cataracts, was documented in this family. NCOA6 is a multifunctional protein, a ubiquitously expressed coactivator of specific nuclear receptors and several other transcription factors [25]. NCOA6 is a large protein consisting of 2063 amino acids, with two nuclear receptor boxes (containing the characteristic LXXLL sequence), two transactivation domains, a dimerization region and an inhibitor region at the *C*-terminal rich in serine, threonine and leucine (STL region). Previous studies targeting the knockout of *Ncoa6* resulted in embryonic lethality between E8.5 and E12.5 [26,27,28,29], which elucidates the crucial role of *Ncoa6* in embryonic development, cell survival and homeostasis. In general, coactivators of the nuclear receptor play a crucial role in modifying the chromatin structure and directly interact with components of Transcription factor II D (TFIID) basal transcription machinery [30].

The expression of the *N*-terminal nuclear receptor box of NCOA6 (that contains a LXXLL1 motif) in postmitotic transgenic mouse lens fiber cells depicted impaired lens fiber cell differentiation and denucleation, resulting in p53-dependent and independent apoptosis [31]. The *N*-terminal nuclear receptor box acts in a dominant negative manner that abolishes the function of NCOA6, depicting a series of lens fiber cell-specific differentiation defects, which includes the disruption of crystallin expression, incomplete and prolonged apoptosis and the inhibition of denucleation in the lens fiber cells. Defects such as persisting nuclei (karyolysis defects) were also observed in the lens-specific knockout of *Ncoa6*. Further, the lens vesicle at E11.5 is normal, indicating that Ncoa6 may not be required either at the initial stages of lens development or lens cell lineage specification. Furthermore, this knockout mouse did not exhibit any morphologically detectable phenotype until three to four weeks of age [31].

In the present study, for the first time, a variation (Gly597Asp) was identified in *NCOA6* associated with human congenital cataracts, along with hearing and speech impairments. The alteration of hydrophobic glycine with acidic aspartic acid might affect the contact sites between amino acids, thereby leading to impaired structures with defects in the interactions with its targets. Interestingly, a previous study on NCOA6 indicated that the amino acids 586−860 readily interact with Thyroid hormone nuclear receptor α (TRα) and Thyroid hormone nuclear receptor β (TRβ)/ ligand-binding domain (LBD) [32]. TRα and TRβ are known to play a crucial role in ear development. The variation G597D identified in the present study is in this region, though predicted to be benign by some of the insilico tools, we speculate that this could be a modifier allele and affect downstream targets. It is therefore tempting to speculate that the variation G597D might interfere with the interaction between NCOA6 and TRα / TRβ, thereby causing hearing impairment in the affected subjects. However further analysis through functional characterization of this specific variation both in vitro and in vivo might elucidate the suggested mechanism. From the literature targeted NGS approach with detailed clinical investigation has proven to be successful in underpinning the molecular mechanism of cataractogenesis. We also emphasize that prognosis of known cataract mutations in extended family members and their future generations could aid in the better management of the disorder.

## 4. Materials and Methods

### 4.1. Patients and Families

Eleven families diagnosed with congenital cataracts were included in this study. Among them, five were retrospective families recruited during 1998–2010. Clinical diagnosis was based on manual examination by the physician in children and slit lamp examination for grownups and adult cooperative patients. The study protocol was in accord with the ethical guidelines of the 1975 Declaration of Helsinki and was approved by the Institutional Ethical Committee, Madras Medical College, Chennai (Approval No. 29022013, dated 25th February 2013). Informed consent for genetic testing was sought and obtained from all patients, parents and family members.

### 4.2. DNA Extraction and Quantification

Genomic DNA was extracted from blood by standard extraction procedures (Phenol Chloroform and Isoamylalcohol (PCI) method). DNA quantification was performed using the Nanodrop (Life technologies, Darmstadt, Germany).

### 4.3. Whole-Exome Sequencing and In Silico Analysis

WES was performed in-house using Illumina NextSeq500; libraries were generated using Nextera Rapid Capture Exome (Illumina) to generate 150-bp paired end reads. Alignment of sequence reads, indexing of the reference genome, variant calling and annotation were done with a pipeline based on Burrows-Wheeler Aligner (BWA) using Base Space Onsite (Illumina). Variants were annotated using Alamut-Batch and visualized on Alamut Viewer 2.2 (Interactive Bio software, Rouen, France). A filtering pipeline was established to identify the most likely disease-causing variant. The variants are sorted out based on already known cataract genes listed in cat map database [33]. Since CCs are rare in the population, only variants with a frequency below 1% were chosen. Frequencies of identified variants were also cross-verified on Exome Sequencing Project (ESP) data and Exome Aggregation Consortium (ExAC) data. Mutations that have been previously described to be disease-causing in the Human Gene Mutation Database (HGMD) and literature were given the highest priority. The effects of mutations that were described in the HGMD were further validated by reading published literature reporting the variants, and only those variants that had convincing evidence were chosen. In addition, protein truncation mutations such as nonsense and frame shift (insertions or deletions) were also ranked higher in priority. Variants were categorized following standardized American College of Medical Genetics (ACMG) criteria [34]. Variants were evaluated for the PP3 in-silico prediction (around 21 known insilico tools embedded in the database) criterion using predictions from Varsome [35].

### 4.4. Primer Design, PCR Amplification and Sanger Sequencing

To confirm the putative variants and their co-segregation among family members, primers were designed using online tools and browsers (UCSC Genome browser, Ensemble) and were standardized in gradient PCR. DNA samples of patients and their family members were used to amplify appropriate gene fragments and sequenced commercially to identify mutations and their co-segregation. The list of primers used in the current study for the confirmation of mutations observed were as given in Supplementary Table S3. Direct sequencing was done at GATC, Konstanz, following the standard procedures, and sequences were BLAST-aligned for homology and variations. All data relevant to the study are included in the article or uploaded as supplementary information. The raw data that support the findings of this study are also available from the corresponding author upon reasonable request.

## 5. Conclusions

In conclusion, the present study demonstrated the efficacy of NGS methods (whole-exome sequencing) in the genetic diagnosis of inherited congenital cataracts. Whole-exome sequencing (WES) was done on 11 families with congenital/childhood cataracts, and the success rate was 55% (six out of 11 families screened). Four novel mutations were observed in already known candidate genes (FYCO1, E979* (ACR12); ΔQ1430 (BCC23), EPHA2, C262Y; P3H2, Q473GfsX19 and TDRD7, V1005YfsX4); one known mutation (G78V) in the PAX6 gene was also documented. A further analysis of the WES data led to the identification of variation in novel gene (NCOA6, G597D) to be associated with congenital cataract and hearing and speech impairment in BCC23 family, which requires further functional validation. Furthermore, the present study also offers a lead scope for a subset of future prospective studies that might aid in the dissection of molecular pathology of certain pathogenic gene variants towards the prognosis and therapeutic intervention of congenital cataracts.

## Figures and Tables

**Table 1:** Summary of Clinical findings in resolved Bilateral Childhood Cataract Cases of the present study.

Case Code	Age at Onset (Years)	POI	Sex (M/F)	Phenotype LE (Left Eye)	Phenotype RE (Right Eye)	Visual Acuity		Other Phenotypic Features if Any	Secondary Affected Relatives	Blood Samples Collected
						LE	RE			
DKEC4	SB	AR	F	Posterior Sub capsular	Posterior Sub capsular	PL (NIP)	PL (NIP)	Grand father’s brother is infertile	Grandfather’s Brother	Proband, Mother, Father, Maternal Grandmother
C343	SB	AD	F	Anterior Polar Cataract with Micro cornea	Anterior Polar Cataract with Micro cornea	NPL	NPL	Aniridia	Paternal Grand mother, Uncles, Aunt, Father, Cousin brothers, Cousin sister	8 Family members (6 affected and 2 unaffected) – Refer Pedigree
CCE13	SB	AR	M	Lamellar cataract + Micro cornea + Nystagmus	Lamellar cataract + Micro cornea + Nystagmus	PL	PL	Nil	Sibling	Proband, Mother, Sibling
CCE27	SB	AR (PH)	-	Patchy, Total Cataract	Patchy, Total Cataract	1/60-BCVA-6/60	6/36-BCVA-6/24	Mother had milder developmental cataract phenotype	Sibling	Proband, Mother, Father, Sibling
ACR12	2.5	AR (PH)	M	Lamellar Cataract	Posterior Cortical Cataract	1/60-NIP	1/60-NIP	Nil	Brother	Proband, Brother, Father, Mother
BCC23	SB	ND (PH)	M	Dense Cortical cataract	Posterior sub capsular	PR	PR	Mother—Blue dot opacities; Proband and Sib—Deaf and Dumb	Two sibs, Mother	Two sibs and Father

Summary of the clinical findings in this study. SB—Since Birth, POI—Pattern of Inheritance; AR—autosomal recessive, AD—autosomal dominant, ND—Not Determined, PH—Phenotypic Heterogeneity, M—Male, F—Female, PL—Perception of Light, NPL—No Perception of Light, NIP—No Improvement with Pinhole, BCVA—Best Corrected Visual Acuity, PR—Faulty PR—Patient is not able to perceive light from the nasal and temporal quadrant.

**Table 2:** Summary of the genetic findings in in resolved Congenital cataract families.

Family-ID	POI	Consanguinity	Zygosity	Gene Variant	Genomic Co-Ordinate	Variant Identified	RS ID and Frequency	ACMG Criteria	Solved Cases
DKEC4	AR	Y	HOM	*TDRD7*	chr9-100249550-A-	TDRD7(NM_014290.3): c.3012delA (p.Val1005TyrfsTer4)	NA-Novel variant not reported before	PATH	S
C343	AD	N	HET	*PAX6*	chr11-31823275-C-A	PAX6(NM_001310158.2) c.233G > T (p.Gly78Val)	rs121907920-No frequency reported	LP	S
CCE13	AR	Y	HOM	*EPHA2*	chr1-16474911-C-T	EPHA2(NM_004431.5): c.785G > A (p.Cys262Tyr)	NA-Novel variant not reported before	VUS	S
CCE27	AR	Y	HOM	*P3H2*	chr3-189692382--C	P3H2(NM_018192.4): c.1417dupG (p.Glu473GlyfsTer19)	NA-Novel variant not reported before	PATH	S
ACR12	AR	Y	HOM	*FYCO1*	chr3-46007891-G-A	FYCO1(NM_024513.4): c.2935C > T (p.Gln979Ter)	NA-Novel variant not reported before	PATH	S
BCC23	ND (PH)	Y	HOM	*FYCO1*	chr3-45965219-CTC	FYCO1(NM_024513.4): c.4288_4290delGAG (p.Glu1430del)	rs774142616-gnomAD-0.0000239/ ISB Kaviar3-0.0000257	VUS	S
		Y	HET	*NCOA6*	chr20-33338208-C-T	NCOA6(NM_014071.5): c.1790G > A (p.Gly597Asp)	rs1461340387-gnomAD-0.0000119	LB	PS

Summary of the genetic findings in this study. POI—Pattern of Inheritance, ND—not determined, AR—autosomal recessive, AD—autosomal dominant, PH—Phenotypic Heterogeneity, Y—Yes, N—No, HET—heterozygote, HOM—homozygote, NA—Not Applicable, ACMG American College of Medical Genetics, PATH—pathogenic, LP—Likely pathogenic, VUS—Variant of uncertain significance, LB—Likely Benign, S solved, PS plausibly solved.

## Supplementary Materials

Supplementary Materials can be found at https://www.mdpi.com/1422-0067/21/24/9569/s1. Table S1: Population frequency of the rare variant in the gene FYCO1(NM_024513.4):c.4288_4290del documented in family BCC23 (Source Genome Aggregation Database (gnomAD exomes), successor to ExAC Database), Table S2: Population frequency of the rare variant in the gene NCOA6:c.1790G>A documented in family BCC23 (Source Genome Aggregation Database (gnomAD exomes)), Table S3: Primers used to check the co-segregation of putative variants documented in Whole exome sequencing.
