# Supplementary material for: Application of WES towards Molecular Investigation of Congenital Cataracts: Identification of Novel Alleles and Genes in a Hospital-Based Cohort of South India
Source: Int J Mol Sci. 2020 Dec 16;21(24):9569. doi: 10.3390/ijms21249569 (PMC7765966; doi:10.3390/ijms21249569)
Supplement: Supplementary file 1 [file ijms-21-09569-s001.pdf]

Supplementary Table S1: Population frequency of the rare variant in the gene FYCO1(NM_024513.4):c.4288_4290del documented in family BCC23 (Source Genome Aggregation Database (gnomAD exomes), successor to ExAC Database).

Population	Allele Count	Allele Number	Homozygotes	Allele Frequency
African	-	16,256	-	-
Ashkenazi Jewish	-	10,080	-	-
East Asian	-	18,394	-	-
European (Finnish)	-	21,648	-	-
European (Non-Finnish)	-	113,762	-	-
Latino	-	34,592	-	-
South Asian	5	30,616	-	0.000163
Other	1	6,140	-	0.000163
Total	6	251,488	-	0.0000239
Male	5	135,918	-	0.0000368
Female	1	115,570	-	0.00000865

Supplementary Table S2: opulation frequency of the rare variant in the gene NCOA6:c.1790G > A documented in family BCC23 (Source Genome Aggregation Database (gnomAD exomes))

Population	Allele Count	Allele Number	Homozygotes	Allele Frequency
African	-	16,252	-	-
Ashkenazi Jewish	-	10,078	-	-
East Asian	-	18,390	-	-
European (Finnish)	-	21,648	-	-
European (Non-Finnish)	-	113,574	-	-
Latino	-	34,584	-	-
South Asian	3	30,616	-	0.0000979
Other	-	6,136	-	-
Total	3	251,278	-	0.0000119
Male	-	135,838	-	-
Female	3	115,440	-	0.0000259

Supplementary Table S3: Primers used to check the co-segregation of putative variants documented in Whole exome sequencing

Primer	Sequence (5'- 3')	Ta (°C)	Amplicon Size(bp)
FYCO1-Ex-8-F	CACCCACTGACAATGAAGCC	66.1	902
FYCO1-Ex-8-R	CCCACAGTACATTCTCCAGC		
FYCO1-Ex-17-F	TCAGCCTGTCCTGCGAGACC	72	872
FYCO1-Ex-17-R	CAGTGGCAGAGCAGTGGGAATC		
NCOA6-Ex-9-F	GTAAGCAGTTATCCCACAGCC	67.6	1176
NCOA6-Ex-9-R	CAACAATGGGCTCGTTAGCG		
P3H2-Ex-9-F	GTCCTATGAGATGCCCAGTC	60	674
P3H2-Ex-9-R	TAGATGGCTGTTGGGCTAGG		
PAX6-Ex-7-F	AGCTCTCTACAGTAAGTTCTC	60	533
PAX6-Ex-7-R	AAAGGAGACAAATGTGGAGC		
EPHA2-Ex-3-F	TCGGACCTGGACTACGGCACC	60	671
EPHA2-Ex-3-R	CATGAGCCACCGTGCCCAGC		
TDRD7-Ex-16-F	CTATCAAATAAGACAGTGGC	57	572
TDRD7-Ex-16-R	CAGCAGATATAATGATAGAGC		
CRYBB1-Ex-6-L	CAGGCACCGTGACCATGCTC	69	691
CRYBB1-Ex-6-R	CTTGACGGCAGGAACTTGGC		
